# Evaluating the effectiveness of video cases to improve patient-centeredness in psychiatry: a quasi-experimental study

**DOI:** 10.5116/ijme.5d9b.1e88

**Published:** 2019-10-25

**Authors:** Kamilla Pedersen, Andreas Bennedsen, Berit Rungø, Charlotte Paltved, Anne Mette Morcke, Charlotte Ringsted, Ole Mors

**Affiliations:** 1 Centre for Health Sciences Education, Faculty of Health, Aarhus University, Aarhus, Denmark; 2 Psychosis Research Unit, Aarhus University Hospital Psychiatry, Denmark; 3 Corporate HR, MidtSim, Central Denmark Region, Aarhus, Denmark; 4 Copenhagen Academy for Medical Education and Simulation at Rigshospitalet, Capital Region of Denmark, Copenhagen, Denmark

**Keywords:** Video cases, medical students, patient-centredness, teaching, psychiatry

## Abstract

**Objectives:**

To evaluate the effectiveness of including interactive video-based patient cases in preparatory lectures on medical students’ patient-centredness and attitudes towards psychiatry.

**Methods:**

This study was designed as a quasi-experimental intervention study. A preparatory lecture on diagnostic interviewing was given to 204 fourth-year medical students before a 4-week psychiatry clerkship. The students were divided into two groups. One group (n=102) received a preparatory lecture including an interactive video case portraying a doctor performing a diagnostic interview with a simulated patient (intervention group). The other group (n=102) received a conventional preparatory lecture using text-based instructional material (control group). We conducted a paired sample t-test to compare the students’ confidence in exhibiting patient-centred communication and their attitudes towards psychiatry before receiving the preparatory lecture and after having completed a minimum of three weeks of clerkship training.

**Results:**

A total of 102 students, 51 in each group, completed a questionnaire at both measurement points. In the intervention group, we found a statistically significantly difference for the students’ patient-centredness before (M=69.4, SD=10.0) and after (M=73.8, SD=8.6) the intervention t_(97)_=2.38, p= 0.02, but no changes in attitudes t_(98) _=1.07, p=0.28. In the control group, we found no changes in patient-centredness or attitudes.

**Conclusions:**

Video cases in preparatory lectures appear to be better than text-based material at improving students’ patient-centredness in psychiatry. However, neither video cases nor text-based material seem to influence the students’ attitudes.

## Introduction

Patient-centredness is considered to enhance clinicians’ understanding of the patient perspective and of their own reactions. A better understanding will allow them to better identify and solve clinical problems when encountering and communicating with psychiatric patients.^1^ Patient-centred communication has been shown to entail higher patient satisfaction, which is associated with improved healthcare, better symptom resolution and even lower mortality rates.^2^ Although a majority of patients favour patient-centred communication, some patients prefer the biomedical approach to solving their problems through “doctor-centred” decision-making.^3^ However, the biomedical approach may exclude the patient from the decision-making process. This could compromise treatment as patients might feel misunderstood or unheard.^3^^,^^4^ An evidence-based structure of the diagnostic interview may combine the biomedical history with the patient’s perspective.^5^

The diagnostic interview in psychiatry is recognised as a unique tool combining the humanistic and the scientific approach to provide a better understanding of the patient.^6^^,^^7^ Like the general public, medical students may see psychiatric patients as dangerous, violent and unlikely to recover.^8^ These attitudes are likely to stem from stereotypical representations of the psychiatric patient in the media and in movies.^9^ Such attitudes may compromise students’ learning about psychiatry and influence their approach to managing psychiatric patients.^8^Moreover, medical students’ emotional challenges in the encounter with psychiatric patients may compromise the quality of communication and diagnostic accuracy when performing a diagnostic interview.^10^ Effective training strategies in patient-centred communication could thus be central to the teaching on the diagnostic interview in clinical psychiatry.^11^

### Literature review

The first psychiatric patients seen by medical students are likely to be patient cases described in textbooks.^12^ Nonetheless, text-based material is not considered optimal for teaching communication as it fails to convey insight into real patients’ lives and emotions and may thus not enhance patient-centredness.^12^^,^^13^To improve medical students’ attitudes towards psychiatry and to encourage patient-centred communication, it has been suggested to present an image of clinical psychiatry as a field that offers positive role models and considers various aspects of patients’ lives and emotions.^14^^,^^15^Role play using trained simulated psychiatric patients and experienced instructors has proven effective in teaching medical students basic and patient-centred communication within psychiatry.^16^ However, such simulation-based teaching formats are resource-demanding set-ups.^17^ As an alternative to the less engaging text-based cases and the costly simulated patient set-up, video cases portraying psychiatric patient cases have been suggested as a tool to enhance students’ preclinical insight before encountering real patients.^15^^,^^18^The effects of using teaching formats including video cases have been widely studied in various undergraduate training programs. The effects have been measured immediately after the teaching in psychiatric training, for example in terms of the medical students’ knowledge and assessment of suicide risk^19^, assessment of ethical sensitivity^20^, attitudes towards psychotherapeutic techniques^21^ and perceived dangerousness of psychiatric patients.^22^ Video cases using simulated psychiatric patients in interactive teaching formats have been suggested to stimulate the patient-centred communication when performing the diagnostic interview^23^^,^^24^, but little is known about their long-term effects on medical students’ patient-centredness and attitudes towards psychiatry.^25^

From the perspective of Bandura’s theory on self-efficacy and behavioural changes, self-protective behaviour is, once established, difficult to change as consistent avoidance will prevent a person from learning that real-life conditions have changed.^26^Likewise, although based on misconceptions,  medical students’ potential fear and insecurity of how to meet psychiatric patients may be difficult to alter.^23^ According to Bandura, exposure to visualised representations of feared situations may mitigate anxiety reactions and reduce avoidance behaviour.^26^ Thus, video cases showing the clinical encounter with the psychiatric patient may serve to expose students to potentially feared clinical situations, e.g. the mental state examination (MSE), and thereby change their approach to and behaviour towards the patients.

On basis on the previous studies findings, of the potentials in using visualized representations of clinical encounters for teaching purposes, we hypothesized that medical students in their psychiatric clerkship taught by video cases portraying clinical practice in psychiatry, would score better in their self-perceived patient-centred approaches and attitudes toward psychiatric patients, than the students receiving a conventional lecture using a text-based material. Thus, in this study, we aimed to evaluate the effectiveness of including interactive video-based patient cases as part of the curriculum in medicine to ensure higher patientcentredness in psychiatry and possibly change their attitudes towards psychiatry.

## Methods

### Study design

A quasi-experimental design was used to evaluate the effectiveness of using video cases. We applied a quantitative survey approach and included a large group of medical students to investigate the effect of using either video cases (intervention group) or conventional text material (control group) on the students’ patient-centredness and their attitudes towards psychiatry.

### Setting and participants

An existing 4-week psychiatry clerkship for fourth-year medical students enrolled at Aarhus University in the Central Denmark Region formed the setting of this study. In the spring of 2016, 204 medical students entered their psychiatry clerkship; these students were eligible for inclusion in this study. Participation in the study was voluntary and students’ identities remained anonymous. The study results did not affect students’ grades and were used for research purposes only. According to Danish law, this type of study did not require approval from the Danish National Committee on Health Research Ethics as no biomedical intervention was performed.

### Sampling procedure

Students are routinely allocated into four clinical clerkships, without regard to sex, age or special requests, across nine psychiatric units located at six psychiatric hospitals in the Central Denmark Region. We applied a quantitative survey approach to a convenience sample of 204 fourth-year medical students. Students were randomly divided into two groups, each of 102 participants, but no other formalised randomisation process was conducted as the study was performed in the existing rotation groups. Three psychiatric lectures, each of two hours’ duration, prepared the fourth-year students to perform diagnostic interviews with psychiatric patients. We conducted an intervention in one of these preparatory lectures in one of the groups by replacing the traditional text-based material with video cases in two of the four rotations.

### Intervention group

The intervention group received lectures including video cases. A senior psychiatrist presented the video case in the classroom-based two-hour preparatory lecture to demonstrate clinical practice as portrayed in the videos and to initiate and facilitate reflection and discussion among students. Students were encouraged to participate interactively by voting (raising a coloured sheet) on response options following the built-in questions in the video.

The video cases portraying the communication and interaction between a simulated patient and a doctor in an acute psychiatric ward formed the basis of the teaching format. The purpose of the video cases was to prepare students for similar tasks during their clinical psychiatry clerkship. The teaching format was designed to cover three important mental conditions that students are likely to encounter in clerkship: mania, schizophrenia and severe depression with psychotic symptoms. The videos depicted both the patients’ and the doctors’ faces and body language. Each video included 8-12 built-in questions on psychopathological symptoms; observations of the portrayed patients’ language, affect, behaviour and the doctor’s approach. The questions were followed by feedback on right/wrong choices with respect to psychopathology and symptoms relevant to the continued diagnostic interview. Each case was supplemented with written examples of how to formulate the information obtained in the diagnostic interview in a patient record. The video cases were delivered via an e-learning platform introduced in the classroom setting by a senior psychiatrist lecturer.

### Control group

The group receiving conventional lectures served as control group. The lecture consisted of a PowerPoint presentation shown by a senior psychiatrist in the classroom-based two-hour preparatory lecture. The slides provided text-based formulations and explanations of psychopathology as the basis for instruction and demonstrations of how to perform the diagnostic interview.

### Evaluation of the results of the intervention

The students’ patient-centredness was evaluated using the 27-item Self-Efficacy in Patient-Centeredness Questionnaire (SEPCQ-27), developed by Zachariae and colleagues in 2015^27^. The SEPCQ-27 has been found to have preliminary construct validity^27^, indicating its usefulness as a measure in patient-centred communication in teaching and research. The SEPCQ-27 questionnaire used in this study was built on Bandura’s work on self-efficacy, as medical student self-efficacy in patient-centredness was defined as his or her confidence in own ability to exhibit a particular behaviour in a patient-centred manner. The questionnaire includes three underlying self-efficacy factors: 1) exploring the patient perspective (items 1-10), 2) sharing information and power (items 11-20) and 3) dealing with communicative challenges (items 21-27). The SEPCQ-27 comprises 27 statements of self-efficacy, and responses are rated on a 5-point Likert scale with endpoints anchored as “To a very low degree” and “To a very high degree”. The score range is 27-135. A mean score of 64.2 has previously been demonstrated for patient-centredness in 448 Danish medical students at the start of their fourth-year (clinical medical/surgical clerkship), and 70.1 has been demonstrated as a mean score for 127 Danish medical students who had completed a course in communication and peer supervision at the end of their third-year clinical clerkship.^27^ The SEPCQ-27 is available in both an English and a Danish version.

The students’ attitudes were measured using the 30-item Attitudes Towards Psychiatry questionnaire (ATP-30), developed by Burra and colleagues in 1982.^28^ The ATP-30 can be used to determine changes in medical students’ attitudes towards psychiatry in relation to teaching. The ATP-30 comprises statements about psychiatry in relation to eight related attitudinal themes: 1) psychiatric patients, 2) psychiatric illness, 3) psychiatrists, 4) psychiatric knowledge, 5) psychiatric career choice, 6) psychiatric treatment, 7) psychiatric institutions and 8) psychiatric teaching. Responses are provided on a 5-point Likert scale from “strongly disagree” (1) to “strongly agree” (5). The ATP-30 includes both positive and negative statements, and the ATP score is the sum of the total scores for both positively and negatively phrased items (minimum score: 30, maximum score: 150). Scores above 90 indicate a favourable attitude, scores below 90 an unfavourable attitude, and a score of 90 a neutral attitude. The ATP-30 has been used in various studies worldwide^29^. Consequently, we found a Danish version of the ATP-30 an appropriate tool for measuring and exploring changes in attitudes in relation to the effects of the video cases in this study, and we performed the translation into Danish.

### Translation procedure: ATP-30

We followed the forward-backward translation and cross-cultural adaption method suggested by Beaton and colleagues.^30^ In stage one, the main author translated the Canadian English version into Danish. An independent expert and another author (individually) translated the English version into Danish. In stage two, we compared the three translations and synthesised them into a single Danish version of the ATP-30 after reaching consensus. Subsequently, we conducted semi-structured pilot test interviews with nine fourth-year medical students, two psychiatric nurses engaged in teaching and two university staff members doing research in educational health science. These interviews focused on wording, translation, relevance, understanding and time consumption. The results of the pilot test interviews were used to modify the existing version, and a pre-final Danish version of the ATP-30 was produced. In stage three, two English native-speakers made a back-translation into English from the pre-final Danish version. In stage four, the two versions were compared and tested against the original version; this did not lead to any alterations. In stage five, we pilot-tested the pre-final Danish version on randomly selected groups of medical students, psychiatric nurses and university staff (n=32); this was done to ensure that the electronic distribution, administration and reminder procedure worked well and that the obtained data output was reliable.

### Data collection

During the mandatory course at the beginning of the first introductory lecture, students were asked to complete the questionnaire, consisting of the SEPCQ-27 and the ATP-30 combined with a total of 57 items, using a link in their student email or a QR code provided at the lecture (pre-intervention questionnaire). The students completed the questionnaires again after having completed a minimum of three weeks of their clinical clerkship training (post-intervention questionnaire). A total of three email reminders were sent during the students’ fourth week of clerkship and the following two weeks to ensure a high response rate.

We measured the medical students’ perception of their own confidence in exhibiting patient-centred communication and their attitudes towards psychiatry. Measures were gathered before and three weeks into their clerkship. The SEPCQ-27 and the ATP-30 questionnaires were distributed at the beginning of the first introductory lecture and were completed by the students. All questionnaires were collected immediately after completion. Participation in the study was voluntary and anonymous. The students completed the questionnaires again after having completed a minimum of three weeks of their clinical clerkship training. Questionnaires were matched by an anonymous student ID number. The data were collected by the web-based survey tool SurveyXact.

### Data analysis and statistics

Inspired by previous studies evaluating self-efficacy and attitudes on the basis of educational interventions,^27^^,^^31^^,^^32^ we chose to use a paired sample t-test to investigate differences in patient-centredness and attitudes between the two groups before the introductory lectures and after completing a minimum of three weeks of clinical clerkship training. P values of less than 0.05 were considered to be significant. The relationship between outcomes was analysed by Pearson’s correlation. All data were analysed using Stata statistical software, version 14.

### Inclusion and exclusion criteria

**Figure 1:**
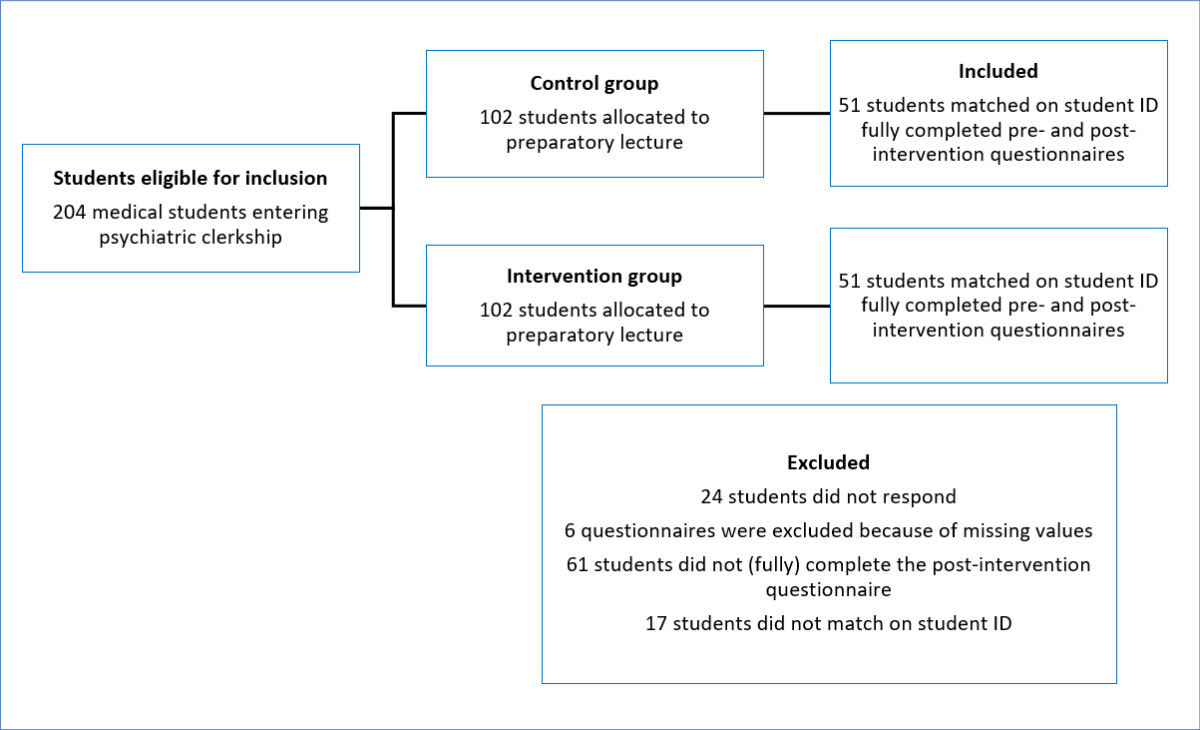
Flowchart of study inclusion

We included only data from fully completed questionnaires. A total of 102 students were excluded from the study because of missing data. The total mean score of missing data for students completing the pre-intervention questionnaire but not the post-intervention questionnaire (n=61) did not differ significantly from the included sample. To evaluate the influence of sample size, the effect size was calculated. The difference was found to be 0.28, which can be characterised as small to moderate.Figure 1 depicts a flowchart of students included in the study.

## Results

A total of 102 students, 51 in each group, completed the SEPCQ-27 and the ATP-30 questionnaires at both measurement points.The ATP-30 scores obtained after clerkship were positively correlated with the SEPCQ-27 scores in both groups (control group: r = 0.289, n = 51, p = 0.039063; intervention group: r = 0.4211, n=51, p = 0.00209).

### Self-efficacy in patient-centeredness

The students’ confidence in exhibiting patient-centred communication use was found to be higher in the group offered video cases (intervention group) than in the group offered text-based instructional material (control group), as shown in Table 1. A significant increase was seen in the SEPCQ-27 mean scores in the intervention group (pre-intervention: M =69.4, SD=10.0; post-intervention: M=73.8, SD=8.6; medical students: t_(97)_ =2.38, p=0.02).

**Table 1:** Total score (mean) for SEPCQ-27 in both groups

Test	Intervention group (video-based teaching material)			Control group (text-based teaching material)		
	pre-intervention clerkship	post-Intervention clerkship	p value	pre-Intervention clerkship	post-Intervention clerkship	p value
SEPCQ-27	69.4 (10.0)	73.8 (8.6)	0.02	71 (10.6)	73.6 (12.5)	0.27

### Attitudes towards psychiatry

No significant increase was seen in the ATP-30 mean scores in the intervention group. pre-intervention: M=104.3, SD= 11.5; post-intervention: M=106.9, SD=12.8; medical students: t_(98) _= 1.07, p= 0.28).

**Table 2:** Total score (mean) for ATP-30 in both groups

	Intervention group (video-based teaching material)			Control group (text-based teaching material)		
Test	pre-intervention clerkship	post-intervention clerkship	p value	pre-intervention clerkship	post-intervention clerkship	p value
ATP-30	104.3 (11.5)	106.9 (12.8)	0.28	105.9 (11.3)	105.5 (16.4)	0.90

## Discussion

This study demonstrated that medical students exposed to video-based patient cases during their preparatory lectures significantly improved their patient-centredness, but no changes were seen in their attitudes towards psychiatry. The medical students exposed to text-based patient cases during their preparatory lectures demonstrated no changes in neither patient-centredness nor attitudes.

### 

### Comparison with existing literature

Our results suggest a positive correlation between students’ attitudes and self-efficacy after their clerkship. However, the association is likely to be influenced by a variety of complex and unidentified factors, for example, exposure to stories in academia about psychiatry^33^, the students’ previous experience with psychiatry^34^ and the students’ emotional engagement with the teaching format.^35^The construct of video cases, in contrast to text-based material, has the ability to convey emotion in the learner.^13^ Video cases used to prepare first- and second-year students on how to meet and manage psychiatric patients have previously been found to influence students’ emotional reactions and thus their learning experiences and perceptions.^36^ As early patient contact is regarded as a precursor to practicing and prepares students for clinical work^37^, the use of video cases may expose students to an emotionally challenging preclinical situation that provides with them tools to manage similar real-life situations ^37^. In contrast, using text-based material to prepare students for clinical work has been suggested to cause a form of emotional detachment and to obstruct their development of a caring and empathic attitude.^13^As both SEPCQ-27 and ATP-30 scoreswere high in the control group from the onset of the study, the students taught with video cases did not have higher SEPCQ-27 end score in the measurement of patient-centredness than the students taught with text-based cases. A focus in future studies could this be to explore the degree to which video cases may influence the patient-centredness of students with either high or low baseline mean scores in the SEPCQ-27.

Preclinical exposure to psychiatric patients in the form of video cases may resemble the use of simulation-based formats and simulated patients in clinical psychiatric teaching, which has also been shown to lead to improved communication.^15^ A recent review on self-efficacy in medical students concluded that self-efficacy is a key factor in student learning and achievement in clinical practice.^38^Students with confidence in their patient-centred communication will be more likely to engage in tasks that require those approaches and more likely to overcome obstacles and challenges.^38^Correspondingly, we found an association between providing students with preclinical experience using video cases and their self-efficacy in terms of patient-centredness; this could suggest improvement of their learning and achievement in the course of their clerkship training.

**Figure 2:**
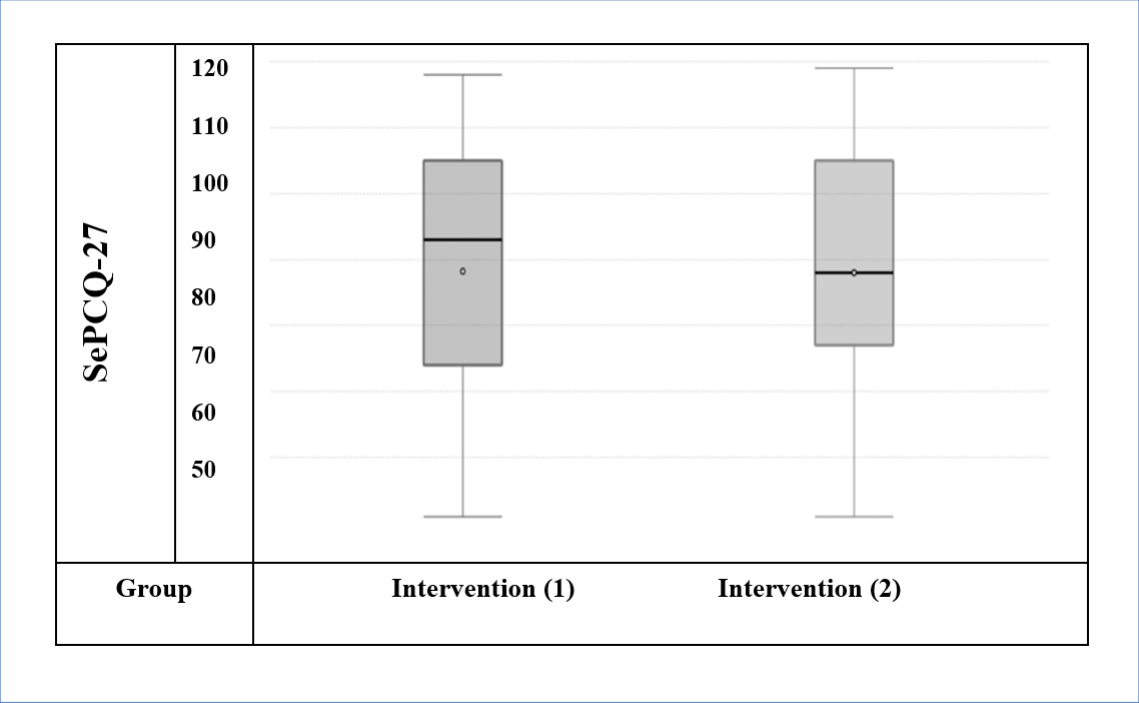
Box plots illustrating the differences between intervention group (1) and control group (2)

### Limitations

The patient-oriented approach to psychiatric teaching in communication has been proposed to be instrumental in improving medical students’ attitudes towards psychiatry.^39^ However, despite the significantly improved scores on self-efficacy in exhibiting patient-centred communication in the intervention group, we found no changes in the students’ attitudes towards psychiatry. One reason could be that favourable attitudes towards psychiatry were found in both the intervention group and the control group, both before and after their clerkship, with mean scores over 90 in the ATP-30. Our findings resonate with international studies using the ATP-30, which also concluded that medical students generally demonstrate positive attitudes towards psychiatry.^40^Another reason for the lack of change in attitudes could be the specific components of the psychiatry clerkship. Recently, a number of contextual clerkship factors, such as interpersonal skills training related to patient management,^41^ educational models like student-run clinics to increase medical students’ perceived knowledge and comfort with psychiatry^42^, and enthusiastic and positive clinical staff encouraging the students’ social integration^43^,have been proposed as influential in furthering students’ long-term positive attitude towards psychiatry. Thus, efforts to improve attitudes towards psychiatry may require more than just one two-hour lecture.

Among the limitations of this study, we recognise that the findings may be difficult to generalise as all students were enrolled at the same institution. The number of students participating in this study was comparable to that of other study populations using the ATP-30 in a pre-post design. Yet, the ATP-30 may be insufficient as a tool for exploring the influence of preparatory lecture formats on students’ attitudes without considering other clerkship factors and their influence on students’ learning. Investigating contextual clerkship factors may serve to provide a deeper understanding of this issue. Another limitation may be found in the interpretation of results from the SEPCQ-27, as the total post-intervention end mean score was similar in the two groups; this could imply that the difference at baseline was the true cause of the significant change. Reduced response rates on the post-intervention questionnaires and missing data led to a smaller sample size, which reduced the statistical power of the study. Only 50% of the students completed both the pre-intervention questionnaire and the post-intervention questionnaire, and this could also have influenced the results if the missing group of students represented other attitudes to patient-centredness. One could hypothesise why students could be more or be less motivated to fill out a questionnaire; this could be related to the perceived relevance or interest in the topic, but we do not know the reasons for the missing data. If we had used just one questionnaire, or a shorter and simpler questionnaire, we might have been able to reduce the number of missing data.

## Conclusions

Our findings suggest that including interactive video cases on the diagnostic interview in preparatory lectures may have a positive effect on medical students’ patient-centredness during their psychiatry clerkship. The ability of video cases to change students’ attitudes towards psychiatry remains unclear.

### Acknowledgments

This study was funded by Aarhus University, the Central Denmark Region, and the Advisory Group for Postgraduate Medical Education North, Denmark.

### Conflict of Interest

The authors declare that they have no conflict of interest.
